# The differences in the anatomy of the thoracolumbar and sacral autonomic outflow are quantitative

**DOI:** 10.1007/s10286-024-01023-6

**Published:** 2024-02-25

**Authors:** Thomas J. M. Verlinden, Wouter H. Lamers, Andreas Herrler, S. Eleonore Köhler

**Affiliations:** 1 Department of Anatomy & Embryology, Faculty of Health, Medicine and Life Sciences, Maastricht University, Universiteitssingel 50, 6229 ER Maastricht, The Netherlands; 2 Tytgat Institute for Liver and Intestinal Research, Academic Medical Center, University of Amsterdam, Amsterdam, The Netherlands

**Keywords:** Autonomic nervous system, Ganglion, Neural crest cells, Neuron, Preganglionic, Postganglionic

## Abstract

**Purpose:**

We have re-evaluated the anatomical arguments that underlie the division of the spinal visceral outflow into sympathetic and parasympathetic divisions.

**Methodology:**

Using a systematic literature search, we mapped the location of catecholaminergic neurons throughout the mammalian peripheral nervous system. Subsequently, a narrative method was employed to characterize segment-dependent differences in the location of preganglionic cell bodies and the composition of white and gray rami communicantes.

**Results and Conclusion:**

One hundred seventy studies were included in the systematic review, providing information on 389 anatomical structures. Catecholaminergic nerve fibers are present in most spinal and all cranial nerves and ganglia, including those that are known for their parasympathetic function. Along the entire spinal autonomic outflow pathways, proximal and distal catecholaminergic cell bodies are common in the head, thoracic, and abdominal and pelvic region, which invalidates the “short-versus-long preganglionic neuron” argument.

Contrary to the classically confined outflow levels T1-L2 and S2-S4, preganglionic neurons have been found in the resulting lumbar gap. Preganglionic cell bodies that are located in the intermediolateral zone of the thoracolumbar spinal cord gradually nest more ventrally within the ventral motor nuclei at the lumbar and sacral levels, and their fibers bypass the white ramus communicans and sympathetic trunk to emerge directly from the spinal roots. Bypassing the sympathetic trunk, therefore, is not exclusive for the sacral outflow. We conclude that the autonomic outflow displays a conserved architecture along the entire spinal axis, and that the perceived differences in the anatomy of the autonomic thoracolumbar and sacral outflow are quantitative.

**Supplementary Information:**

The online version contains supplementary material available at 10.1007/s10286-024-01023-6.

## Introduction

The universally accepted model of the sympathetic and parasympathetic efferent limbs of the autonomic nervous system was formulated at the turn of the nineteenth to the twentieth century [1]. In addition to physiological and pharmacological criteria, anatomical arguments have been invoked to define the sympathetic-parasympathetic model [2–4]. These anatomical arguments have three main components. The first argument relates to the bimodal distribution of peripheral cell bodies, with the sympathetic cell bodies located in ganglia close to the central nervous system, and the parasympathetic cell bodies in a distal position, within or close to the wall of target organs. A second argument involves the absence of white rami communicantes at the sacral level. In contrast to the thoracolumbar sympathetic outflow, preganglionic neurons at the sacral level bypass the sympathetic trunk. Pelvic splanchnic nerves arise, therefore, directly from the sacral plexus. The third argument concerns the gap in the autonomic outflow at the lumbar level. As the autonomic outflow is concentrated around the T1-L2 and S2-S4 levels, the cell bodies of the preganglionic neurons do not appear as a continuous cell column. A parallel is therefore often drawn between the parasympathetic cranial and sacral outflows [2].

In this review, we re-evaluate the anatomical arguments that divide the spinal visceral outflow in sympathetic and parasympathetic partitions. A systematic literature search permitted us to map the location of catecholaminergic neurons throughout the entire mammalian peripheral nervous system. Subsequently, a narrative method was employed to characterize segment-dependent differences in the location of preganglionic cell bodies and the composition of white and gray rami communicantes. In total, it becomes apparent that the differences between the thoracolumbar and sacral outflow are not binary. The anatomy of the autonomic outflow displays a conserved architecture along the entire spinal axis, albeit with a quantitative gradient in characteristic features. This finding is compatible with recent data indicating that the molecular signature of preganglionic cells in the thoracolumbar and sacral region is highly similar [5].

## Methods

This study meets the Preferred Reporting Items for Systematic Reviews and Meta-Analyses guidelines (see Supplemental Word document and Supplemental interactive Tables 1 and 2). Literature was searched for studies dealing with the anatomical arguments that are used to divide the spinal visceral outflow into sympathetic and parasympathetic divisions. Using a systematic literature search, we first mapped the location of catecholaminergic neurons throughout the entire mammalian peripheral nervous system. The outcome of the systematic literature prompted us to look more closely into the location of the preganglionic neurons and the white and gray rami communicantes along the spinal cord. This search followed a narrative strategy and included studies from the nineteenth and twentieth centuries. We ensured that only findings that comply with current scientific understanding are included in this review. Languages were restricted to English, German and French.

**Table 1:** List of nerves containing catecholaminergic neurons

Nerve	First author, year, and reference	Species	Extra signal
Oculomotor	Oikawa, 2004 [198]	Human	
	Maklad, 2001 [15]	Mouse	
	Ruskell,1983 [199]	Monkey	
Trochlear	Hosaka, 2014 [76]	Human	
	Oikawa, 2004 [198]	Human	
	Maklad, 2001 [15]	Mouse	
Trigeminal, ciliary, submandibular, pterygopalatine, otic, trigeminal ganglion	Teshima, 2019 [78]	Mouse, human	#, *(Hand2+)
	Hosaka, 2016 [200]	Human	
	Matsubayashi, 2016 [201]	Human	
	Yamauchi, 2016 [77]	Human	#
	Hosaka, 2014 [76]	Human	#
	Szczurkowski,2013[72]	Chinchilla	#
	Kiyokawa, 2012 [69]	Human fetus	#
	Rusu, 2010 [206]	Human	
	Thakker, 2008 [68]	Human	#
	Kaleczyc, 2005 [67]	Pig	#, *(DBH+)
	Reynolds, 2005 [24]	Rat	#, *(DBH+)
	Maklad, 2001 [15]	Mouse	
	Grimes, 1998 [66]	Rhesus monkey	#, *(DBH+)
	Kirch, 1995 [64]	Human	#
	Ng, 1995 [75]	Rat, monkey	#
	Tan, 1995 [65]	Cat, monkey	#
	Simons, 1994 [71]	Rat	#, *(DBH+)
	Marfurt, 1993 [23]	Rat, guinea pig	#
	Tyrrell, 1992 [63]	Rat	#
	Shida, 1991 [74]	Rat	#
	Soinila, 1991 [73]	Rat	#
	Yau, 1991 [213]	Cat	
	ten Tusscher, 1989 [214]	Rat	
	Kuwayama, 1988 [70]	Rat	#
	Landis,1987 [61]	Rat	#, *(DBH+)
	Uemura,1987 [62]	Japanese monkey, cat, dog	#
	Jonakait, 1984 [49]	Rat (embryo)	#
	Lackovic, 1981 [166]	Human	*(NA+)
Abducens	Oikawa, 2004 [198]	Human	
	Maklad, 2001 [15]	Mouse	
	Lyon,1992 [202]	Cynomolgus monkey	
	Johnston, 1974 [203]	Human	
Facial, geniculate ganglion	Tereshenko, 2023 [204]	Human	
	Tang, 2022 [25]	Mouse	#
	Ohman-Gault, 2017 [205]	Mouse	
	Matsubayash, 2016 [201]	Human	
	Yamauchi, 2016 [77]	Human	
	Hosaka, 2014 [76]	Human	
	Reuss, 2009 [207]	Rat	
	Maklad, 2001 [15]	Mouse	
	Johansson, 1998 [208]	Rat	
	Shibamori, 1994 [14]	Rat	
	Takeuchi, 1993 [209]	Cynomolgus monkey	
	Fukui, 1992 [210]	Cat	
	Anniko, 1987 [167]	Mouse	*(NA+)
	Matthews, 1986 [13]	Cat	
	Wilson, 1985 [12]	Cynomolgus, rhesus monkeys	
	Thomander, 1984 [211]	Cat	
	Schimozawa, 1978 [212]	Mouse	
Vestibulocochlear, vestibular ganglion	Yamauchi, 2016 [77]	Human	
	Shibamori, 1994 [14]	Rat	
	Hozawa, 1993 [155]	Guinea pig	*(DBH+)
	Yamashita, 1992 [215]	Guinea pig	
	Hozawa, 1990 [154]	Cynomolgus monkey	*(DBH+)
	Anniko, 1987 [167]	Mouse	*(NA+)
	Paradiesgarten, 1976 [216]	Cat	
	Densert, 1975 [168]	Rabbit and cat	*(NA+)
Glossopharyngeal, petrosal ganglion	Oda, 2013 [79]	Human	#
	Ichikawa, 2007 [37]	Rat	#
	Matsumoto, 2003 [217]	Rat	
	Wang, 2002 [36]	Rat	#
	Satoda, 1996 [220]	Cynomolgus monkey	
	Ichikawa, 1995 [34]	Rat	#
	Ichikawa, 1993 [33]	Rat	#
	Helke, 1991 [32]	Rat	#
	Helke, 1990 [29]	Rat	#
	Katz, 1990 [30]	Rat	#
	Kummer, 1990 [31]	Guinea pig	#
	Katz, 1987 [28]	Rat	#
	Katz, 1986 [27]	Rat	#
	Jonakait, 1984 [49]	Rat (embryo)	#, *(DBH+)
	Katz, 1983 [26]	Rat	#
Vagus, superior and inferior (nodose) ganglion	Bookout, 2021 [47]	Mouse	*(DBH+, Hand2+)
	Verlinden, 2016 [80]	Human	#, *(DBH+)
	Hosaka, 2014 (75)	Human	
	Seki, 2014 [218]	Human	
	Onkka, 2013 [219]	Dog	
	Ibanez, 2010 [82]	Human	#
	Kawagishi, 2008 [38]	Human	#
	Ichikawa, 2007 [37]	Rat	#
	Matsumoto, 2003 [217]	Rat	
	Nozdrachev, 2003 [221]	Cat	
	Forgie, 2000 [222]	Mouse	
	Yang, 1999 [150]	Rat	#, *(DBH+)
	Gorbunova, 1998 [45]	Rabbit	
	Ichikawa, 1998 [35]	Rat	#
	Sang, 1998 [46]	Mouse	#
	Ichikawa, 1996 [43]	Rat	#
	Uno, 1996 [44]	Dog	#
	Fateev, 1995 [223]	Cat	
	Ichikawa, 1995 [34]	Rat	#
	Zhuo, 1995 [42]	Rat	#
	Zhuo, 1994 [41]	Rat	#
	Yoshida, 1993 [40]	Cat	#
	Ruggiero, 1993 [228]	Rat	
	Dahlqvist, 1992 [81]	Rat	#, *(DBH+)
	Helke, 1991 [32]	Rat	#
	Helke, 1990 [29]	Rat	#
	Kummer, 1990 [31]	Guinea pig	#
	Ling, 1990 [230]	Hamster	
	Baluk, 1989 [232]	Guinea pig	
	Katz, 1987 [28]	Rat	#
	Dahlqvist, 1986 [171]	Rat	*(NA+)
	Lucier, 1986 [235]	Cat	
	Matthews, 1986 [13]	Cat	
	Smith, 1986 [237]	Guinea pig	
	Blessing, 1985 [238]	Rat	
	Smith, 1985 [240]	Guinea pig	
	Jonakait, 1984 [49]	Rat (embryo)	#, *(DBH+)
	Katz, 1983 [26]	Rat	#
	Hisa, 1982 [241]	Dog	
	Lackovic, 1981 [166]	Human	*(NA+)
	Ungváry, 1976 [243]	Cat	
	Nielsen, 1969 [169]	Cat	*(NA+)
	Kummer, 1993 [39]	Rat	#
	Lundberg, 1978 [242]	Cat, Guinea pig	
Accessory	Hosaka, 2014 [76]	Human	
Hypoglossal	Hosaka, 2014 [76]	Human	
	Tubbs, 2009 [83]	Human	#
	Tseng, 2005 [224]	Hamster	
	Tseng, 2001 [225]	Hamster	
	Hino, 1993 [226]	Dog	
	Fukui, 1992 [210]	Cat	
	O’Reilly, 1990 [227]	Rat	
Greater auricular	Matsubayashi, 2016 [201]	Human	
Phrenic	Verlinden, 2018 [120]	Human	*(DBH+)
	Lackovic, 1981 [166]	Human	*(NA+)
Suprascapular	Hosaka, 2014 [76]	Human	
Mammary	Eriksson, 1996 [229]	Human, rat	
Lateral antebrachial cutaneous nerve of forearm (musculocutaneous)	Marx, 2011 [231]	Human	
	Marx, 2010 [233]	Human	
Radial	Marx, 2010 [234]	Human	
Superficial branch of radial	Marx, 2010 [234]	Human	
	Marx, 2011 [231]	Human	
Palmar branch of ulnar	Balogh, 1999 [236]	Human	
Medial antebrachial cutaneous nerve of forearm	Marx, 2011 [231]	Human	
	Marx, 2010 [239]	Human	
Intercostal	Lackovic, 1981 [166]	Human	*(NA+)
Genitofemoral	Lackovic, 1981 [166]	Human	*(NA+)
Ilioinguinal	Lackovic, 1981 [166]	Human	*(NA+)
Sciatic	Creze, 2017 [149]	Human fetus	
	Hosaka, 2014 [76]	Human	
	Loesch, 2010 [147]	Rat	
	Castro, 2008 [146]	Rat	
	Wang, 2002 [244]	Mouse	
	Li, 1999 [245]	Rat	
	Li, 1996 [247]	Rat	
	Li,1995 [248]	Rat	
	Li, 1994 [250]	Rat	
	Koistinaho, 1991 [249]	Human fetus	
	D’Hooge, 1990 [174]	Dog	*(NA+)
	Studelska, 1989 [254]	Rat	
	Dahlström, 1987 [173]	Rat	*(NA+)
	Dahlström, 1986 [172]	Rat	*(NA+)
	Larsson, 1986 [165]	Rat	*(DBH+, NA+)
	Schmidt, 1984 [164]	Rat	*(DBH+)
	Larsson, 1984 [163]	Rat	*(DBH+, NA+)
	Evers-Von Bültzingslöwen, 1983 [162]	Rabbit	*(DBH+)
	Dahlström, 1982 [170]	Rat	*(NA+)
	Jakobsen, 1981 [161]	Rat	*(DBH+)
	Häggendal, 1980 [160]	Rat	*(DBH+)
	Reid, 1975 [159]	Rat	*(DBH+)
	Keen, 1974 [157]	Rat	*(DBH+, NA+)
	Nagatsu, 1974 [158]	Rat	*(DBH+)
	Dairman, 1973 [156]	Rat	*(DBH+)
	Thoenen, 1970 [266]	Rat	
Fibular	Tompkins, 1985 [148]	Human	
	Jänig, 1984 [246]	Cat	
	Ben-Jonathan, 1978 [175]	Cat	*(NA+)
Tibial	Koistinaho, 1991 [249]	Human fetus	
Sural	Fang, 2017 [251]	Rabbit	
Pudendal	Nyangoh Timoh, 2017 [252]	Human fetus	
	Bertrand, 2016 [253]	Human fetus	
	Hinata, 2015 [255]	Human	
	Hieda, 2013 [256]	Human	
	Alsaid, 2011 [257]	Human fetus	
	Alsaid, 2009 [258]	Human fetus	
	Roppolo, 1985 [259]	Monkey	
Perineal	Moszkowicz, 2011 [260]	Human fetus	
	Colombel, 1999 [261]	Human	
Nerve to levator ani	Hinata, 2014 [262]	Human	
	Hinata, 2014 [263]	Human	
Pelvic splanchnic	Jang, 2015 [18]	Human	#
	Imai, 2006 [17]	Human	#
	Takenaka, 2005 [16]		
Spinal root, dorsal root ganglia	Massrey, 2020 [99]	Human	#
	Morellini, 2019 [269]	Rat	*(DBH+, NAT+)
	Oroszova, 2017 [59]	Rat	#
	McCarthy, 2016 [58]	Mouse	#
	Brumovsky, 2012 [57]	Mouse	#
	Li, 2011 [56]	Mouse	#
	Dina, 2008 [55]	Rat	#, *(DBH+, NAT+)
	Brumovsky, 2006 [54]	Mouse	#
	Ichikawa, 2005 [53]	Mouse	#
	Holmberg, 2001 [52]	Mouse	#
	Deng, 2000 [264]	Rat	
	Jones, 1999 [265]	Rat	
	Ma, 1999 [267]	Rat	
	Shinder, 1999 [268]	Rat	
	Thompson, 1998 [270]	Rat	
	Karlsson, 1994 [271]	Rat	
	Vega, 1991 [51]	Rat	#
	Kummer, 1990 [31]	Guinea pig	#
	Katz, 1987 [28]	Rat	#
	Price, 1985 [50]	Rat	#
	Jonakait, 1984 [49]	Rat (embryo)	#, *(DBH+)
	Price, 1983 [48]	Rat	#
	Lackovic, 1981 [166]	Human	*(NA+)

For each nerve, studies confirming tyrosine hydroxylase-positive nerve fibers are listed with first author, year of publication and species investigated. Studies that demonstrate catecholaminergic (CA) cell bodies are indicated by #. Studies that demonstrate additional “sympathetic” phenotypic properties are indicated by *. DBH: dopamine β-hydroxylase, NA: noradrenaline, NAT: noradrenaline transporter. A more extensive interactive Microsoft Excel-based Table, provided with filter tools for study characteristics and findings, is provided in supplemental interactive Table 1

### Data source and study selection for the systematic literature search

Abstracts, titles, and Medical Subject Headings (MeSH) entry terms in PubMed were searched to identify original studies that either established the existence of catecholaminergic neurons histologically by demonstrating the presence of the enzymes tyrosine hydroxylase or dopamine β-hydroxylase, or confirmed communication between nerves and sympathetic structures using validated techniques such as neural tract tracing, experimental neural degeneration, crushing or denervation, and neural recording. Studies relying on macroscopic dissections alone were not included. Search terms included every nervous structure listed in *Terminologia Anatomica* [Anatomical Terminology] [6] under the headings “cranial nerves,” “spinal nerves” and “parasympathetic part of autonomic part of peripheral nervous system.” The last search was performed on July 11, 2023. The reference lists of retrieved articles were also reviewed for additional studies that fulfilled the search criteria.

### Search strategy for the systematic literature search

The search for each structure consisted of two separate approaches. The first approach looked for the histologically confirmed presence of catecholaminergic neurons, and was executed by combining the entry terms of tyrosine hydroxylase or dopamine β-hydroxylase with (query term: *AND*) the nervous structure of interest. The second approach searched for communication between nerves and sympathetic structures, using the entry terms of sympathetic structures listed in *Terminologia Anatomica* [6] *AND* the nervous structures from search 1, *AND* neuroanatomical tract-tracing techniques (MeSH) OR horseradish peroxidase (MeSH) OR communication OR communicating OR communications OR anastomosis OR anastomosing OR connecting OR connection.

## Findings and Discussion

For the systematic approach, a total of 43 queries for the cranial and 101 for the spinal nerves were performed, which provided information on 389 anatomical structures in 996 and 243 identified studies, respectively. All abstracts were screened for the inclusion criteria with respect to applied techniques, language, and species, resulting in 170 eligible studies (Table 1 and supplemental interactive Table 1 [extended Microsoft Excel-based Table]).

The narrative approach produced 60 relevant references from the nineteenth and twentieth centuries. Supplemental interactive Table 2 provides an overview of the findings extracted from these studies. Although the scientific views put forward in these studies often no longer meet current models, they do frequently present research findings that were made with still accepted techniques. The recent molecular studies of the development of cranial ganglia from Schwann cell precursors and their source [7–9], for instance, were preceded by specific histological observations in the early twentieth century [10, 11]. These classical observations have the advantage of including human embryos.

### The distribution of catecholaminergic neurons

#### Nerve fibers

Throughout the mammalian body, catecholaminergic nerve fibers have been demonstrated in many spinal and all cranial nerves and ganglia (Table 1). Catecholaminergic nerve fibers are also present in established parasympathetic nerves [4, 6], such as the greater petrosal nerve in mice, rats, cats, and monkeys [12–15] and the pelvic splanchnic nerves in humans [16–18]. We found no species-specific differences. Although we focused on mammals, we also encountered similar observations in birds [19, 20] and amphibia [21, 22], suggesting evolutionary conservation of the observed features.

#### Cell bodies

Catecholaminergic cell bodies have a more widespread distribution than generally acknowledged [2, 4, 6]. They are found in the trigeminal [23, 24], geniculate [25], inferior glossopharyngeal [26–37], superior [31, 38], and inferior [26, 28, 29, 31, 32, 34, 35, 37–47] vagal ganglia, and in dorsal root ganglia at all spinal levels [28, 31, 48–59]. Moreover, the generally accepted parasympathetic ganglia of the head [2], which include the ciliary [60–69], the otic [69], the pterygopalatine [69–72] and the submandibular ganglia [69, 73–78], all contain catecholaminergic cell bodies. Catecholaminergic cell bodies are found not only in ganglia, but also in the cranial nerves themselves, such as the (lingual branch of the) glossopharyngeal nerve [27, 79], the cervical [80] and laryngeal branches of the vagus nerve [81, 82], and the cranial root of the hypoglossal nerve [83]. In addition, catecholaminergic cell bodies are found in both the ventral and dorsal spinal nerve roots [84–99], and in the hypogastric and the (“parasympathetic”) pelvic splanchnic nerves [16, 17].

### Argument 1: The short versus long preganglionic neuron

**Fig. 1:**
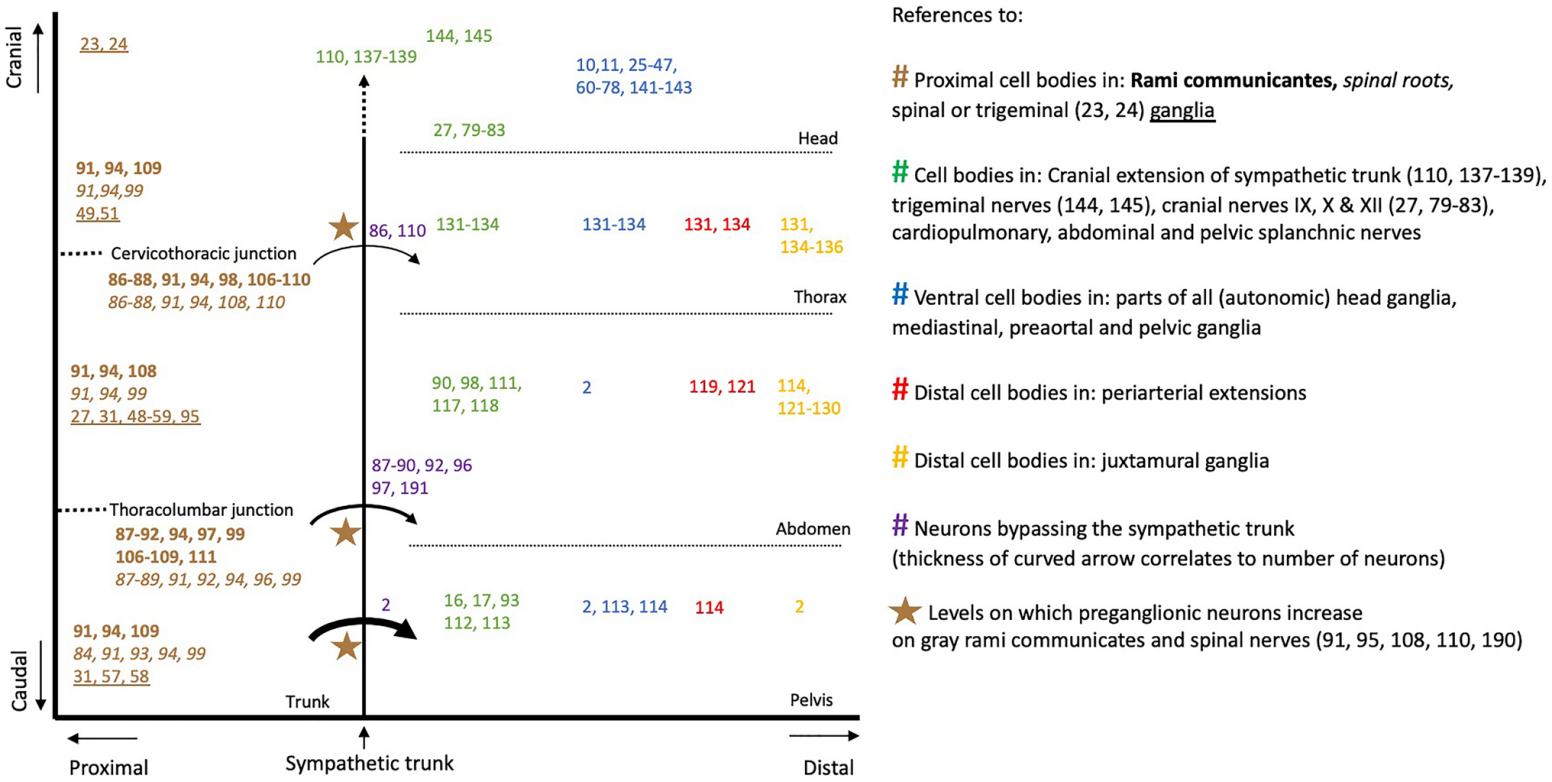
Definitive catecholaminergic cell positions. References are plotted showing the position of cell bodies along the cranio-caudal (Y) and proximo-distal (X) body axes. Altogether, the data show that both proximal and distal ganglia are common in the entire thoracolumbar and sacral autonomic outflow pathways. Other references indicate the levels at which preganglionic neurons bypass the sympathetic trunk (curved arrows), or more frequently use the gray rami communicantes (brown stars)

A commonly held concept in the classic subdivision of the autonomic outflow is the bimodal distribution of cell bodies, with the sympathetic cell bodies located in ganglia close to the central nervous system, and the parasympathetic cell bodies in a distal position, within or close to the wall of target organs. Our systematic review, in contrast, demonstrates that both proximal and distal catecholaminergic cell bodies are common throughout the entire spinal outflow (Fig. 1). The distribution of the cell bodies, therefore, cannot be used to subdivide the autonomic outflow.

#### Proximal locations

Catecholaminergic neurons are descendants of neural crest cells [100–102]. Trunk neural crest cells consist of several migrating groups. The core of the developing ganglia is established by an early cohort of neural crest cells that migrate ventrally to the mesenchyme dorsolateral to the dorsal aorta [102–105]. Many of these proximal cell bodies subsequently nest in the sympathetic trunk. Other proximal locations, however, include the spinal nerve roots [84–99], dorsal root ganglia [28, 31, 48–59, 95], and white and gray rami communicantes [85–91, 95–98, 106–111]. In the pelvic area, usually characterized as parasympathetic, these proximal locations of ganglionic cells also exist, both in sacral nerve roots [84, 93, 99] and in the proximal part of the pelvic splanchnic nerves [16, 17, 93, 112–114].

#### Distal locations

Neural crest cells can run aground anywhere along their proximo-distal migration pathways. Cell bodies of trunk neural crest cell origin are found up to the walls of the target organs, as the vascular system keeps instructing these cells to migrate [115, 116]. In the abdomen, cell bodies are found in large numbers in all splanchnic nerves [90, 98, 111, 117, 118], the preaortic ganglia [113, 114], all their periarterial extensions [119, 120], and within the walls of organs of both the urogenital and gastrointestinal tracts [114, 121–130]. In the thorax, the situation is similar. Catecholaminergic cell bodies are found on the cardiopulmonary nerves [131–134], in small mediastinal ganglia [131–134] and the ganglion cardiacum [135, 136], and within the wall of the heart [131, 134]. Finally, the distal position of cell bodies that are of trunk neural crest cell origin extends to the head. Several thousands of cell bodies exist, for example, along the intracranial course of human internal carotid arteries [110, 137–139].

#### (Catecholaminergic) cell bodies within the autonomic ganglia of the head are of mixed origin

The cell bodies within the cranial autonomic ganglia develop from neural crest cells and the related Schwann cell precursors [102, 140, 141], with the majority coming from Schwann cell precursors. Schwann cell precursors that are associated with the oculomotor nerve [8, 11, 141], chorda tympani [7, 10, 11], greater superficial petrosal nerve and geniculate ganglion [7, 10, 11], and tympanic nerve and petrosal ganglion [9–11] populate the ciliary, submandibular, pterygopalatine, and otic ganglia, respectively. Small ganglia are also present along these nerve paths [9–11]. In some species, including humans, at least part of these autonomic ganglia originate directly from cranial neural crest cells. These migrate along the ophthalmic [10, 11, 141–143], maxillary [11, 142], and mandibular nerves [11, 142], and also populate the ciliary, submandibular and pterygopalatine, and otic ganglia, respectively. Similarly, these trigeminal nerve branches also harbor small ganglia that represent grounded cell bodies [144, 145]. In addition, cells from the superior cervical ganglion were shown to populate the otic ganglion [10].

**Fig. 2:**
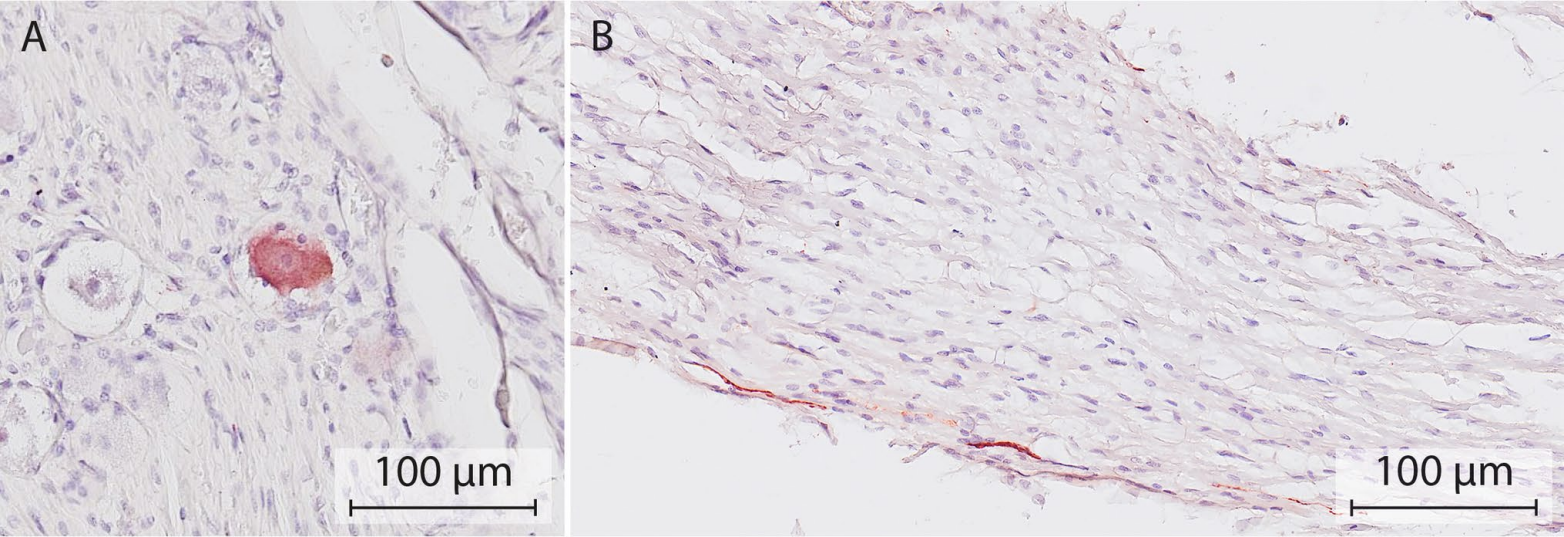
Catecholaminergic neurons in the human geniculate ganglion and the greater superficial petrosal nerve. Example of a TH-positive cell body (**A**) and nerve fiber (**B**) in the geniculate ganglion and proximal course of the superficial petrosal nerve, respectively. Nerve tissue was harvested from a formalin-fixed cadaver (97 years of age) from the body donation program of the Department of Anatomy and Embryology, Maastricht University. The body was preserved by intra-arterial infusion with 10 L fixative (composition (v/v): 21% ethanol, 21% glycerin, 2% formaldehyde, 56% water, and 16 mmol/L thymol), followed by 4 weeks of fixation in 20% ethanol, 2% formaldehyde, and 78% water. Antibody: Abcam ab209487, 1:10,000. Antigen retrieval Tris–EDTA pH 9.0, 30 min. Secondary antibody GAR-bio, 1:10,000. Chromogen: Vector NovaRED peroxidase substrate kit, SK-4805

The partly catecholaminergic phenotype of the autonomic ganglia of the head may arise from cranial neural crest cells and Schwann cell precursors, as catecholaminergic cell bodies are present in both trigeminal [23, 24], and petrosal [26–37] and geniculate ganglia [25], respectively. Thus far, catecholaminergic cell bodies have not been reported to exist in the human geniculate ganglion, but here we demonstrate a few (Fig. 2). Of relevance, TH-positive neurons were also present in the proximal course of the greater superficial petrosal nerve. The notion that the partly catecholaminergic phenotype of the submandibular ganglion may arise from cranial neural crest cells is supported by the finding that catecholaminergic cell bodies in this ganglion are already present prior to the arrival of postganglionic neurons from the superior cervical ganglion [78].

### Caveats of using the catecholaminergic phenotype

In aggregate, our inventory convincingly shows that catecholaminergic fibers and cell bodies are present in the entire tracts of peripheral nerves and ganglia throughout the body. The reported prevalence of cell bodies per location varies greatly (supplemental interactive Table 1) [16, 18, 25, 27, 30–35, 37, 39, 41, 42, 44, 46, 51, 53, 54, 62–67, 71, 72, 74, 80, 122, 124, 126, 146–150]. We hypothesize that the reasons for this variation are both biological and technical. Most studies were not quantitative in design, because such studies would require random sampling of a sufficient number of histological sections across the entire structure of interest and, to deal with biological variation, a sufficient number of independent samples. In addition, the fraction of catecholaminergic cells that stain is influenced by such factors as the quality of the antibodies, the concentration of the neurotransmitter or enzyme, and the time between death and fixation. Even though it remains to be established what functions these cells have, their distribution pattern is too uncommon to dismiss as coincidental.

#### The catecholaminergic phenotype is not always associated with efferent (sympathetic) neurons, nor is it always permanent

Nerve cells with catecholaminergic phenotypic properties arise from the neural crest or the related Schwann cell precursor population. From these progenitors, different functional subtypes develop [151]. The catecholamines dopamine, noradrenaline, and adrenaline are derivates of phenylethylamine [152]. Tyrosine hydroxylase (TH) is the first enzyme in the biosynthetic pathway of (nor)adrenaline. This enzyme has received the most attention in biomedical research [153], and is often, incorrectly, associated with (nor-)adrenergic neurotransmission. Co-localization of TH with dopamine β-hydroxylase (DBH), which catalyzes the β-hydroxylation of dopamine to noradrenaline, provides stronger evidence for such neurotransmission. DBH-positive neurons have been reported in the ciliary [61, 66, 67], pterygopalatine [71], trigeminal [24, 49], petrosal [49], nodose [47, 49, 150], and dorsal root ganglia [49], and the vestibulocochlear [154, 155], vagus [80, 150], recurrent laryngeal [81], phrenic [120], and sciatic [55, 156–165] nerves. Other studies measured concentrations of noradrenaline directly in the trigeminal [166] and nodose [45] ganglia, and in the facial [167], vestibulocochlear [167, 168], vagus [166], phrenic [166], ilioinguinal [166], genitofemoral [166], sciatic [157, 163, 165, 169–174], and fibular [175] nerves and spinal nerve roots [166]. TH-positive, but DBH-negative cell bodies have been observed in ciliary [62, 64, 67], petrosal [31], jugular [31], nodose [31, 39] and dorsal root ganglia [31, 48, 51]. Furthermore, TH-positive, but noradrenaline transporter type-1- [57] and phenylethanolamine-*N*-methyl-transferase-negative cell bodies [31] were reported in dorsal root ganglia. Nerves in which solely TH but none of the downstream enzymes are present probably utilize dopamine as a neurotransmitter.

Tyrosine hydroxylase-positive staining has been observed in cell bodies that exhibit morphological features typical of primary sensory neurons in petrosal [26, 34], nodose [26, 34], geniculate [25], and dorsal root ganglia [56, 57, 59]. Some TH-positive cell bodies in the nodose ganglion are also labeled following the injection of tracer material into the nucleus of the solitary tract [39, 44].

Within the developing ciliary and pterygopalatine ganglia, neurons are observed that express catecholamines transiently [176–178]. In the mouse, the nerve fibers of the vagus nerve arrive in the wall of the gastrointestinal tract only after the TH-positive cells of the vagal neural crest cells have settled there [179, 180]. The TH-positive cells have largely disappeared from the vagus nerve by embryonic day 16 in the mouse, which corresponds to ~9.5 weeks of development in human embryos. Our observations suggest that a subset of these transiently TH-positive cells might remain present.

### Arguments 2 and 3: The absence of white rami communicantes at the sacral level and the “lumbar gap”

Two other anatomical arguments that have been used to define the sympathetic-parasympathetic model are the absence of white rami communicantes at the sacral level, and the gap in the autonomic outflow at the lumbar level.

#### The rami communicantes are part of a peripheral connection matrix

Macroscopic studies of the distribution pattern of the rami communicantes and sympathetic trunk have shown that the rami communicantes form a true mesh, with up to seven rami communicantes connecting the sympathetic trunk with the spinal nerves from corresponding and adjacent levels [2, 94, 98, 181–184]. Interconnecting bundles of nerve fibers between the left and right sympathetic trunk are present at all levels [98, 185]. In addition, the white and gray rami communicantes can share an epineurium, and then present as a single ramus communicans [91].

#### Mixed content of white rami communicantes

The rami communicantes are defined by their macroscopic appearance [186, 187] which, in turn, depends on the proportion of myelinated nerve fibers present. Macroscopically identifiable white rami communicantes are present between vertebral levels T1 and L2 in humans. Accordingly, the absence of white rami communicantes at the sacral level has been one of the anatomical arguments for separating the sacral from the thoracolumbar autonomic outflow [1]. However, the nerve fibers in the rami communicantes represent not only preganglionic neurons, but also somatic neurons [87, 108, 188]. In addition, the white rami communicantes contain a great number of medium-sized and large myelinated afferent fibers, particularly in the lower thoracic region [189]. The number and size of these afferent fibers together far exceed the small myelinated efferent components, so that the white rami communicantes represent the thoracolumbar inflow as much as the outflow [189].

#### Mixed content of gray rami communicantes

Myelinated preganglionic neurons are also common in the gray ramus communicans [91, 95, 110, 190]. At the upper and lower margins of the thoracolumbar outflow, where the white rami communicantes gradually disappear, the number of myelinated nerve fibers in the gray rami communicantes actually increases tenfold [91, 108]. Sporadically, sacral gray rami communicantes are so heavily myelinated that they have been described as a “sacral white ramus communicans” [93].

#### Preganglionic neurons can bypass the rami communicantes at the thoracolumbar outflow margins

**Fig. 3:**
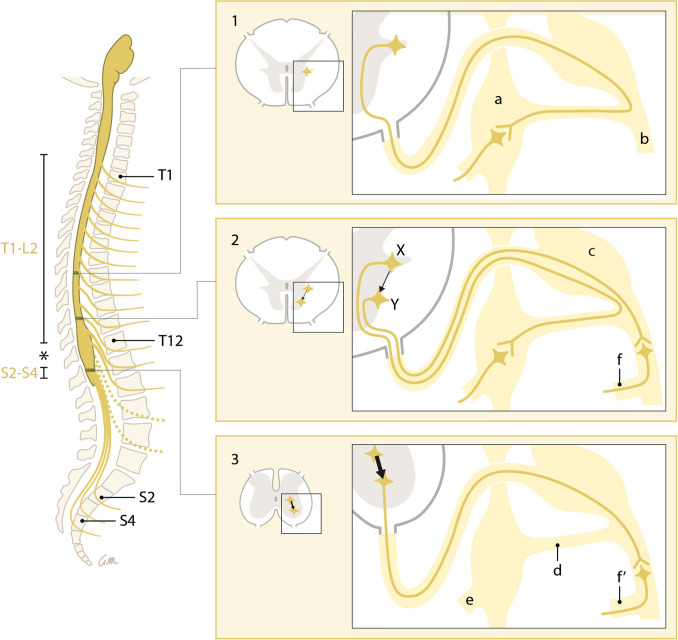
Cranio-caudal change in the position of the cell bodies and the course of the preganglionic neurons. Simplified representation. Left: Preganglionic outflow at the levels T1-L2 and S2-S4. The “lumbar gap” is indicated by an asterisk. Dashed outflow: preganglionic neurons within the “lumbar gap.” Right: From the lower margin of the thoracolumbar outflow downward (panel 1), a gradually increasing number (represented by arrow thickness) of preganglionic neurons originate from cell bodies within or near the ventral motor nuclei and bypass the sympathetic trunk (panels 2 and 3, neuron Y). Bypassing the sympathetic trunk, therefore, is not exclusive for the sacral outflow. Lumbar splanchnic nerves can arise directly from the lumbar plexus (Panel 2, f), such as pelvic splanchnic nerves arise from the sacral plexus (Panel 3, f′). Panels 1 and 2, neuron X: Classic representation of a preganglionic neuron with its cell body in the intermediolateral nucleus. Labels are identical in all panels; a: sympathetic trunk ganglion, b: spinal nerve, c: spinal ganglion, d: rami communicantes, e: splanchnic nerves

At the margins of the thoracolumbar outflow, a fraction of the preganglionic neurons bypass the rami communicantes and the sympathetic trunk [86–90, 92, 96, 97, 110] (Fig. 3). Lumbar splanchnic nerves can arise directly from the lumbar plexus [90, 191], such as pelvic splanchnic nerves arise from the sacral plexus. Bypassing the sympathetic trunk, therefore, is not exclusive for the sacral outflow.

#### The lumbar gap

The spinal preganglionic outflow levels vary among species [1, 84, 92, 112, 186, 192]. In humans, the spinal preganglionic outflow has classically been confined to the segments T1-L2 and S2-S4 based on the absence of white rami communicantes caudal to segment L2, and on the perceived discontinuity of the spinal autonomic outflow cell column. The presence of this “lumbar gap” is often quoted when describing the parallel between the parasympathetic cranial and sacral outflows. Preganglionic neurons have been described, however, at the lower lumbar level [95, 137, 190], which is in the middle of the “lumbar gap.” These preganglionic neurons follow, as described in the previous paragraph, the spinal nerves and gray rami communicantes.

From the thoracolumbar outflow margin downwards, preganglionic cell bodies increasingly nest between the ventral motor nuclei [193, 194] (Fig. 3). At the sacral level, the intermediolateral nucleus no longer forms a distinct lateral horn of gray matter [194], whereas the ventral motor nucleus becomes highly mixed with preganglionic neurons [195]. Apparently, preganglionic neurons with their cell bodies in, or near, the ventral motor horn prefer to follow the path of the motor neurons and branch away towards their targets only distal to the white rami communicantes. At the sacral level, this phenomenon is structural, and the white ramus communicans is absent.

## Conclusion

We conclude that the anatomy of the autonomic outflow displays a conserved architecture along the entire spinal axis, albeit with a gradient in characteristic features. Langley appears to have understood the limitations of the anatomical arguments that support his model, because he acknowledged the nonbinary distribution of postganglionic cell bodies [112, 196], the failure of the white-gray appearance of the rami communicantes to convey their anatomical identity, and the presence of preganglionic fibers in the gray rami communicantes and within the lumbar gap [190]. Although Langley created an appealing concept, his binary model was hallowed by constant repetition in the literature. As a result, the textbook presentation of the division of the spinal visceral outflow in sympathetic and parasympathetic divisions became primarily based on the generalization of this concept.

The conserved architecture of the spinal visceral outflow that is presented in this review seems to be compatible with the finding that the molecular signature of cells in the thoracolumbar and sacral region of the autonomic nervous system is qualitatively highly similar [5, 197].

### Perspective

**Fig. 4:**
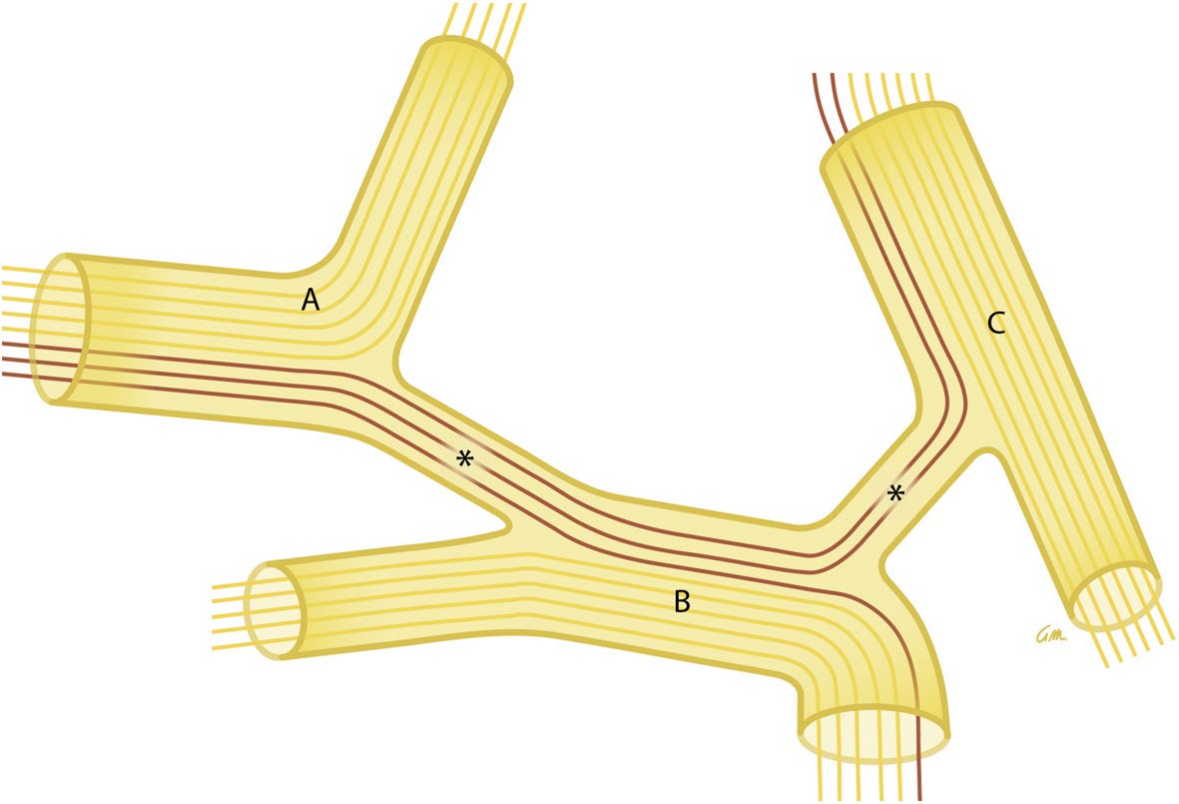
Scheme of neurons using two or more nerves to reach their target organ. Neuronal function is not strictly coupled to specific nerves: neurons change course (purple) from nerve **A** to nerve **B** and **C** via communicating nerve branches (asterisks). This behavior could fit a still hypothetical peripheral connectome

The development of the peripheral nervous system is intricate and diverse. Examples include the reciprocal interaction between neural crest cell migration and nerve formation. It is likely that neural crest cells with catecholaminergic fates emigrate from the CSN along pre-existing nerves to differentiate on their way and/or at their final target site. This might result in the entwined anatomy that we observed. Nerves and ganglia are therefore not homogeneous collections of neurons (Fig. 4). Upon migrating to a distal location, an appreciable number of these catecholaminergic cells apparently strand along the route. Such stranded cells could be very useful as left-behind markers of the migratory routes that were used. It is obvious that the mechanism underlying these cell decisions is of key importance and should be explored experimentally. The involvement of chemotaxis is probably a safe guess. Another intriguing question is whether the features now described for catecholaminergic cells also apply to other populations of nerve cells. If so, the CNS would prove an important source of the migratory nerve cells and the target site or cells an important attractant. It would make the wiring diagram complex and subject worthy of separate study, after identifying the source and target of the neural signals.

### Supplementary Information

Below is the link to the electronic supplementary material.

Supplementary file1 (DOCX 51 KB)

Supplementary file2 (XLSX 32 KB)

Supplementary file3 (XLSX 177 KB)

## Abbreviations

Important note: Nerves and ganglia are *not* homogeneous collections of neurons. Nerves and ganglia themselves therefore are not exclusively sympathetic, parasympathetic, or somatic. For example, the white and gray rami communicantes are not dedicated “sympathetic nerves,” just as the vagus nerves are not exclusively “parasympathetic,” or the spinal ganglia “somatic.”
Neuron: Nerve cell
Cell body (soma, perikaryon): Part of neuron containing the cell nucleus
Nucleus: Collection of cell bodies in the central nervous system
Nerve fiber: Extension of the neuron that propagates the electrochemical stimulus from (axon) or to (dendrite) the cell body
Preganglionic neuron (visceral efferent neuron): Autonomic neuron with its cell body in the central nervous system
Postganglionic neuron: Autonomic neuron with its cell body in the peripheral nervous system
Autonomic outflow (visceral outflow): The group of preganglionic neurons leaving the central nervous system at a certain level
Ganglion: Collection of cell bodies in the peripheral nervous system
Nerve: The nerve fibers and cell bodies that are embedded in connective tissue called epineurium
Nerve branch: Branched extension of a nerve
Sympathetic trunk, also known as paravertebral ganglionic chain: Collection of nerves and ganglia located on either side of the vertebral column
Preaortic (prevertebral) ganglia: Collection of nerves and ganglia located ventral to the abdominal aorta
Pelvic ganglion (inferior hypogastric plexus): Collection of nerves and ganglia located in the pelvic wall
