# Supplementary material for: The differences in the anatomy of the thoracolumbar and sacral autonomic outflow are quantitative
Source: Clin Auton Res. 2024 Feb 25;34(1):79–97. doi: 10.1007/s10286-024-01023-6 (PMC10944453; doi:10.1007/s10286-024-01023-6)
Supplement: Supplementary file 1 — Supplementary file1 (DOCX 51 KB) [file 10286_2024_1023_MOESM1_ESM.docx]

*Search strategy for the systematic literature search*

The search was conducted to the 389 anatomical structures listed in the Anatomical Terminology under the headings ‘cranial nerves’, ‘spinal nerves’ and ‘parasympathetic part of autonomic part of peripheral nervous system’.

The search of each structure consisted of two separate approaches. The first approach looked for the histologically confirmed presence of catecholaminergic neurons, and was executed by combining the entry terms of tyrosine hydroxylase or dopamine β-hydroxylase with *(*query term: *AND)* the nervous structure of interest. The second approach searched for communications between nerves and sympathetic structures, using the entry terms of sympathetic structures listed in the Anatomical Terminology [6] *AND* the nervous structures from search 1, *AND* neuroanatomical tract-tracing techniques (Mesh) OR horseradish peroxidase (Mesh) OR communication OR communicating OR communications OR anastomosis OR anastomosing OR connecting OR connection.

The following figure illustrates the search using the queries (*, **, ***, ****) for the facial nerve as example.


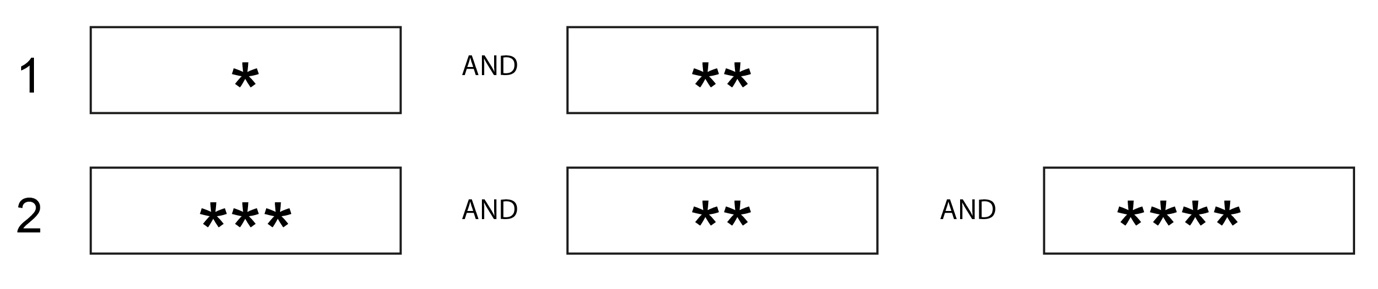


* Tyrosine hydroxylase OR dopamine β-hydroxylase

** Facial nerve (Mesh) OR geniculum OR nerve to stapedius OR posterior auricular nerve OR digastric branch OR stylohyoid branch OR communicating glossopharyngeal OR parotid plexus OR temporal branch OR zygomatic branch OR buccal branch OR lingual branch OR mandibular branch OR cervical branch OR intermediate nerve OR geniculate ganglion OR greater petrosal nerve OR parasympathetic root of pterygopalatine ganglion OR chorda tympani OR parasympathetic root of submandibular ganglion OR communicating tympanic plexus OR communicating vagus

*** sympathetic trunk OR sympathetic ganglion OR cervical ganglion OR thoracic ganglion OR thoracic ganglia OR lumbar ganglion OR lumbar ganglia OR sacral ganglion OR sacral ganglia OR ganglion impar OR cervicothoracic ganglion OR stellate ganglion OR ansa subclavia OR splanchnic nerve OR carotid nerve plexus OR cardiac nerve OR cardiac branch

**** Neuroanatomical tract-tracing techniques (Mesh) OR horseradish peroxidase (Mesh) OR communication OR communicating OR anastomosis OR connecting OR connection
